# Lower Viral Response to Pegylated Interferon Alpha 2a Treatment of Chronic Hepatitis B in Roma People in Eastern Slovakia

**DOI:** 10.1155/2016/8682494

**Published:** 2015-12-29

**Authors:** Sylvia Drazilova, Martin Janicko, Pavol Kristian, Ivan Schreter, Branislav Kucinsky, Marek Kozlej, Ivana Hockickova, Peter Jarcuska

**Affiliations:** ^1^Department of Internal Medicine, Poprad Hospital, Banícka 28, 05845 Poprad, Slovakia; ^2^1st Department of Internal Medicine, Medical Faculty of Pavol Jozef Šafárik University in Košice, Trieda SNP 1, 04001 Košice, Slovakia; ^3^Department of Infectology and Travel Medicine, Medical Faculty of Pavol Jozef Šafárik University in Košice, Trieda SNP 1, 04001 Košice, Slovakia

## Abstract

*Aim*. To evaluate the compliance and virological response to pegylated interferon alpha 2a treatment of chronic hepatitis B in Roma population compared to majority Caucasian population in Slovakia. *Methods*. Retrospective evaluation of a cohort of all Roma patients treated with pegylated interferon alpha 2a from 2007 to 2013 in 3 centers for treatment of chronic viral hepatitis B. The Study included 43 Roma patients with chronic viral hepatitis B and randomly selected control group. Treatment duration was 48 weeks. Viral response was evaluated after 24 weeks, at the end of treatment, and 24 weeks after the end of treatment. *Results*. Complete treatment course was finished by 79.1% of Roma patients compared to all patients from the control group (*p* = 0.0009). There was a tendency toward lower viral response rate in Roma at all time points; however significant difference was only at end of treatment viral response (51.2% Roma versus 81.4% majority, *p* = 0.003). We also did not find significant difference at the rate of HBsAg loss. *Conclusion*. Roma patients with chronic hepatitis B have significantly worse compliance to treatment with pegylated interferon and they have significantly lower rate of end of treatment viral response.

## 1. Introduction

Hepatitis B virus infection remains to be a serious healthcare problem. Chronic viral hepatitis is recognized to be the leading cause of liver cirrhosis worldwide [1]. Around 350–400 million of people worldwide are infected at one time with hepatitis B virus, but more than two billion people are infected with hepatitis B virus during their lifetime [2]. Chronic hepatitis B (CHB) can result in liver cirrhosis and hepatocellular carcinoma, which can occur even in inactive carriers of hepatitis B virus without liver cirrhosis [3]. Every year more than one million people die due to liver failure as a direct consequence of hepatitis B infection [4]. One of CHB treatment options remains pegylated interferon. This therapy not only leads to the improvement of biochemical, virological, and histological findings but also causes twofold reduction of liver cirrhosis risk and 5-fold reduction of hepatocellular carcinoma risk in the average time horizon of 6.8 years [5].

Roma population, due to its peculiarities, poses a significant challenge to the healthcare system. It is estimated that almost two million Roma people live in Central and Eastern Europe alone [6]. Although, due to the significant difference between the official demographic statistics and the estimated count, it is not possible to determine the exact Roma population count, it is reasonable to assume that it represents a substantial population size. Roma population in Eastern Slovakia tends to segregate from the majority population in closed settlements. Their way of living combined with the nonvaccination creates an environment with high risk for hepatitis B infection. In fact, in our previous studies we have determined that the prevalence of hepatitis B in these communities is as high as 12.5% [7]. In this study we wanted to evaluate the treatment effectiveness and compliance in this population and compare it with the majority.

## 2. Methods

This retrospective observational cohort study included all Roma patients with CHB treated with pegylated interferon alpha 2a (PEG IFN) from 2007 to 2013 in three centers for treatment of viral hepatitis (1st Department of Internal Medicine and Department of Infectious Diseases and Travel Medicine, Pavol Jozef Šafárik University in Košice, and University Hospital of L. Pasteur in Košice and Department of Internal Medicine, Poprad Hospital). All treatment was reimbursed by the national healthcare system and was completely free of charge for the recipients. All administrative background was handled by the prescribing physician. Altogether 43 Roma patients were included. Control group consisted of 46 randomly chosen patients from non-Roma (majority Caucasian) population treated with PEG IFN for CHB that were matched by gender. Age matching was impossible because of the lack of patients with appropriate age in the control group. Study was performed in accordance with principles of the Declaration of Helsinki. Institutional Ethics Committee of Pavol Jozef Šafárik University in Košice waived the need for informed consent based on the retrospective nature of the study.

General indications for CHB treatment with PEG IFN in these three centers were observed:

- elevated alanine aminotransferase (ALT) activity in the previous 6 months,
- replication of hepatitis B virus.

Patients were treated with 180 mcg of PEG IFN (Pegasys, Roche, Switzerland) once a week for 48 weeks. HBsAg, HBeAg, and anti-HBs antibodies were tested by Enzygnost (Siemens, Germany) before start of the treatment, at the end of the treatment (M12), and 6 months after the end of the treatment (M18). HBV DNA was tested quantitatively before start of the treatment, 6 months after the start of treatment (M6), at the end of the treatment (M12), and 6 months after the end of the treatment (M18) by Cobas AmpliPrep/Cobas 2.0 (Roche, Switzerland) with the lower limit of detection at 20 IU/mL. Routine biochemical analyses were performed by Siemens Advia 2400 autoanalyzers (Siemens, Germany). Liver cirrhosis was evaluated either histologically or indirectly by liver morphology and clinical signs.

Virological response was present if patients achieved HBV DNA levels <2000 IU/mL at any time point; sustained virological response was present if patients had HBV DNA < 2000 IU/mL 6 months after the end of treatment (EoT). Complete virological response was defined by undetectable HBV DNA and loss of HBsAg [8].

Values are reported as mean ± standard deviation or absolute and relative counts. HBV DNA was transformed using decadic logarithm and reported as median (interquartile range). Continuous variables were compared using Mann-Whitney test. Categorical variables were compared by chi-squared test or Fisher's exact test. Adjusted odds ratios were calculated by multivariate logistic regression. Analyses of virological response for the different time points were performed for per-protocol population. Due to large dropout of Roma patients also real-world effectiveness, which included dropout Roma patients as nonrespondents, was calculated.

## 3. Results

Out of all 43 enrolled Roma patients, 39 remained in the study at M6, 32 remained at EoT (M12), and 29 returned also 6 months after the end of the treatment (M18) for followup. On the other hand, all patients from the control group completed the treatment and 34 out of 46 were available for followup at M18.

**Figure 1:**
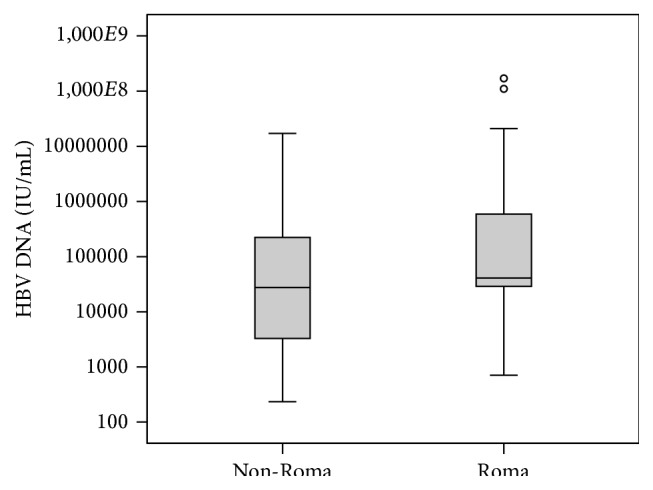
HBV DNA before treatment in Roma and majority (non-Roma) population.

Baseline parameters of the study cohort are reported in Tables 1 and 2. Roma patients had significantly higher HBV DNA levels (Figure 1), but we did not find any difference in the HBeAg positivity, significantly increased ALT, HBV DNA < 2000 IU/mL, or prevalence of liver cirrhosis between Roma and control group. Biopsy was performed in half of the participants, but no difference in average METAVIR score was detected. Also no difference was found in selected biochemical and hematological tests, except the levels of HDL cholesterol (Table 2).

Roma patients had significantly worse compliance to the treatment. All majority patients completed the whole treatment course compared to only 79.1% of Roma patients (*p* = 0.0009). There was also a tendency toward higher loss of followup among Roma patients at M18 compared to control group (32.6% versus 26.1%; *p* = 0.502) (Table 5).

There was a tendency toward lower virological response in Roma patients in all of the specified time points; however per-protocol analysis showed no difference between Roma and control groups in the achievement of virological response at M6, M12, or M18 as well as in the rate of undetectable HBV DNA (Table 3). However, real-world EoT response was significantly more frequent in non-Roma patients compared to Roma patients (80.4% versus 51.2%) (Table 4). This was true even after adjustment for age and sex (OR for Roma 0.148; 95% CI 0.047–0.467). No significant difference was present in the prevalence of virological response at M6 or M18, or the rate of HBV DNA negativization in any of the time points (Tables 3 and 4). All patients who achieved virological response (HBV DNA < 2000 IU/mL) had ALT within normal range at M12 and M18.

There was also a tendency toward lower rates of HBsAg loss and anti-HBs seroconversion in Roma people compared with control group at all time points. However absolute occurrences of both HBsAg loss and anti-HBs seroconversion were very low (less than 4% in Roma and less than 10% in non-Roma population) and the differences were statistically insignificant.

Out of 9 HBeAg positive Roma patients 6 completed the treatment. Three patients lost HBeAg and gained anti-HBe antibodies which lasted up to 6 months after EoT. Two of these patients had HBV DNA at EoT and 6 months after the EoT less than 2000 IU/mL. In the control group only one patient underwent HBeAg seroconversion, but this patient had HBV DNA over 2000 IU/mL at all time points. No patient from both groups who did not lose HBeAg had HBV DNA less than 2000 IU/mL.

In the course of the treatment no serious adverse events or any other important medical events were recorded. After EoT 2 patients from the majority population died, one due to liver failure and one due to abdominal aneurysm dissection.

## 4. Discussion

Chronic hepatitis B can be treated by PEG IFN or nucleot(s)ide analogues (NUC). Interferon based treatment is, compared to NUC treatment, temporary [8]. Based on the published data, 48-week treatment course with 180 mcg PEG IFN once weekly leads to anti-HBe antibody formation in 27% of patients at EoT and 32% of patients at week 24 after EoT with PEG IFN. Decrease of HBV DNA under 100 000 copies/mL occurs in 52% of patients at EoT and 32% of patients 24 weeks after EoT. Decrease of HBV DNA under 400 copies/mL occurs in 25% and 14% of patients at EoT and 24 weeks after EoT, respectively. Loss of HBsAg is sporadic. At week 72 after EoT occurs in 3% of patients, most of these patients have genotype A [9].

In the multicentric study which enrolled HBeAg negative patients treated with PEG IFN in the same dose and duration, HBV DNA decreased under 20 000 copies/mL in 81% at EoT and in 43% it remained under 20 000 copies/mL 24 weeks after EoT. Only in 63% of patients HBV DNA decreased under 400 copies/mL at EoT and in 19% it remained under 400 copies/mL at month 6 after EoT. HBsAg loss occurred in less than 4% of patients [10].

Non-Roma patients in our study achieved similar results; however in Roma patients the results were marginally worse. In every time point there was a trend toward worse outcome of the treatment in Roma population, which achieved statistical significance when the virological response at EoT was evaluated. To our knowledge no study was published in the current literature that would evaluate the efficacy of PEG IFN treatment in the Roma population; therefore we have no data for comparison. Multiple host related factors influence the PEG IFN treatment efficacy. It has been shown that Asian race and genotype A favor the HBsAg seroconversion [10]. Conversely, E genotype that is more prevalent on the African continent is associated with poorer PEG IFN treatment efficacy [11]. Studies that would identify other socioeconomic risk factors for poor PEG IFN treatment efficacy are lacking.

The reasons for worse efficacy of treatment in this particular population are not known. One of the possible causes of this phenomenon is the lower compliance to treatment and lower rate of successfully completed treatment by Roma patients (21% dropout compared to 0% dropout in the control group). Lower socioeconomic standard and other factors associated with segregation in marginalized communities are also possible causes of lower treatment efficacy [12]. Significant portion of Roma patients in our study came from such settlements. In a recent study these people reported worse healthcare availability, higher distrust to medical personnel, and higher anxiety of medical examination. Moreover, they tend to prefer their own alternative treatments compared to the majority population [13].

PEG IFN treatment adherence is not optimal. In the prospective randomized controlled trial by Janssen et al. only 84% completed the treatment (12 are withdrawn for misconduct and 11 discontinued therapy early because of side effects) and further 7 (5.6%) were lost to followup [14]. Real-life studies with posttreatment followup for at least 6 months documented the loss of patients ranging from 3.4% [15] to 20% [16]. In our study, the loss of followup at M18 was 26% in majority population and 37% in Roma population. In a study focused on the long-term NUC treatment of hepatitis B poorer adherence was significantly associated with female sex, younger age, and lower income. This corresponds to our patients because Roma people were significantly younger and poorer.

We can only speculate about other reasons for this reported lower PEG IFN treatment efficacy. The origins of Roma population of Europe have not been confirmed so far, but the prevailing opinion is that they migrated to Europe from India. We therefore performed a search of Web of Science, Medline, and Scopus databases but found no study which evaluated the efficacy of PEG IFN treatment specifically in Indian population. Three small studies reported the efficacy of CHB treatment with recombinant interferon alpha. One randomized study included 20 patients treated for 4 months with recombinant interferon alpha 2b dosed 3 MIU three times weekly for four months. Loss of HBeAg with negative HBV DNA was present in 50% of patients after the end of the treatment and in 65% 12 months after the end of the treatment. Only three patients lost HBsAg as well. CHB relapse was not documented in any of these patients [17]. Another study reported the results of the same treatment regimen in HBeAg positive patients. Mean time of followup was 2.2 years after EoT. Nine out of 14 patients lost HBeAg (64%) and only one patient lost HBsAg as well [18]. In our study Roma people achieved worse results despite the treatment with PEG IFN for 48 weeks. HBeAg seroconversion was present in 50% of Roma who completed the whole treatment course and only one-third of Roma achieved HBeAg seroconversion as well as HBV DNA under 2000 IU/mL.

Third study from India included only anti-HBe positive patients treated with recombinant interferon alpha 2b in the same regimen as reported in previous two studies. At EoT 13 out of 18 patients were HBV DNA negative; however 7 out of these relapsed in the next 12 months after EoT. Relapse was more frequent in cirrhotic patients [19]. In our study, no Roma patient had negative HBV DNA 6 months after EoT. Decrease of HBV DNA under 2000 IU/mL was detected at EoT (M12) in 76.9% anti-HBe positive Roma patients who completed the full treatment course, but only in 47.8% of patients HBV DNA remained low 6 months after EoT. However it is probable that the treatment population in the three mentioned Indian studies was probably highly selected and the efficacy of interferon based treatment in unselected population would be considerably lower.

Another factor that could influence the virological response to treatment with PEG IFN is virus genotype. PEG IFN treatment is more effective in genotypes A and B compared to genotype C or D; moreover, patients with genotype C or D have more unfavorable course of the disease [20, 21]. The relative geographical prevalence of viral genotypes depends greatly on the migration patterns [22]. The most common HBV genotype in Scandinavia, Germany, Czech Republic, Belgium, Hungary, and Poland is genotype A. On the other hand, in Latvia, Estonia, Spain, Italy, Albania, Croatia, Romania, Serbia, Greece, and Russia, the most common genotype is genotype D [23]. Unfortunately the data about the most prevalent genotype in Slovakia are not known. In the Czech Republic the most common is genotype A (67%), followed by genotype D (28%). Only 3% of patients have genotype B and 2% have genotype C. In Hungary the prevalence of VHB genotypes is very similar, with genotype A being the most common (47%), followed by genotype D (43%) and mixed genotypes in 7% of patients [23]. Roma people however are relatively closed ethnic minority with different origins than the majority population. Therefore, the genotypes most prevalent in these people probably differ from the genotypes in the majority population. The probable place of origin of this minority is North/Northwestern India [24]. Most prevalent genotypes in this region are genotypes A and D (46% and 48%, resp.), followed by patients with mixed genotype. Genotype C in this region is practically nonexistent [25]. The most common genotype in East India is genotype D (75%), followed by genotype A (25%). Genotype C is present in 18% of patients and has a higher risk of hepatocellular carcinoma than other genotypes [22]. We are planning to determine the virus genotypes in this population, which could partially explain the differences in reported treatment efficacy.

Hepatitis B prevalence in Roma population in Slovakia is as high as 12.5% [7] and treatment is very expensive. Improvement of socioeconomic conditions, lifestyle, and poverty among Roma people could help to decrease the burden of the disease in this population [26]. The vaccination against hepatitis B in this high risk population could potentially radically decrease the prevalence of the disease and decrease the risk of progression to liver cirrhosis, liver failure, and hepatocellular carcinoma [4]. Vaccine has been discovered in 1971, but nationwide vaccination of newborns against hepatitis B in Slovakia started in 1998 [27, 28]. The prevalence of hepatitis B in this age category is unknown, but it is supposedly lower than that in older age groups. The people born before 1998 are not vaccinated and they are the main reservoir of the infection in the Roma population. Treatment with PEG IFN or NUCs is expensive and adherence in Roma population is poor, which was demonstrated in our study. Optimal solution from the medical as well as economical point of view would be the vaccination of adult patients who have not come into the contact with hepatitis B.

## 5. Conclusion

The results of this study have shown that although the PEG IFN treatment of CHB in Roma population is effective, it is less effective than that in the majority population. More research needs to be done to identify the causes of this phenomenon, although observed lower compliance to treatment is one possible explanation. Substantial combined effort is required to effectively decrease the burden of hepatitis B among Roma people.

## Figures and Tables

**Table 1:** Baseline demographic and virological parameters of the study cohort.

	Roma	Non-Roma	*p*
Count	43	46	
Age (years ± SD)	33 ± 8.3	35 ± 7.5	0.081
Women	11 (25.6%)	13 (28.3%)	0.680
HBeAg posit	9 (20.9%)	4 (8.7%)	0.093
HBV DNA IU/mL (median(IQR))	40900 (28900–588000)	27550 (3300–223367)	0.025
HBV DNA < 2000 IU/mL	3 (9.3%)	7 (15.2%)	0.219
ALT > 2 ULN	22 (51.2%)	21 (45.7%)	0.603
Staging (Metavir)	1.64 ± 1.07	1.34 ± 1.04	0.382
Grading (Metavir)	1.91 ± 0.87	2.0 ± 0.76	0.795
Cirrhosis	3 (7.0%)	5 (10.9%)	0.521

ALT: alanine aminotransferase.

**Table 2:** Baseline biochemical and hematological parameters of the study cohort.

	Roma (mean ± SD)	*n*	Non-Roma (mean ± SD)	*n*	*p*
ALT (*µ*kat/L)	1.52 ± 1.47	43	1.62 ± 1.22	46	0.703
GGT (*µ*kat/L)	0.90 ± 0.64	43	1.06 ± 1.15	46	0.977
TC (mmol/L)	4.90 ± 0.90	43	5.10 ± 0.95	43	0.478
LDL-C (mmol/L)	3.00 ± 0.60	22	3.14 ± 0.87	35	0.605
TAG (mmol/L)	2.37 ± 4.72	27	1.26 ± 0.69	43	0.167
HDL-C (mmol/L)	1.18 ± 0.30	25	1.47 ± 0.36	44	0.003
Bilirubin (*µ*mol/L)	11.95 ± 5.77	43	13.70 ± 6.75	46	0.349
Albumin (g/L)	46.91 ± 4.44	43	45.24 ± 3.55	46	0.176
Platelets (10^9^/L)	201.40 ± 60.05	43	198.82 ± 65.29	45	0.754
WBC (10^9^/L)	6.43 ± 2.28	43	6.03 ± 1.46	45	0.628

ALT: alanine aminotransferase, GGT: gamma-glutamyl transferase, TC: total cholesterol, LDL-C: low density lipoproteins, TAG: triacylglycerols, HDL-C: high density lipoproteins, and WBC: white blood cells.

**Table 3:** HBV DNA during treatment and followup, per-protocol data.

	Roma		Non-Roma		*p*
M6 HBV DNA <2000 IU/mL	26/39	66.7%	37/44	84.1%	0.06
M6 HBV DNA negative	3/39	7.7%	7/44	15.9%	0.172
M12 HBV DNA <2000 IU/mL	22/32	68.8%	37/46	80.4%	0.237
M12 HBV DNA negative	3/32	9.4%	9/46	19.6%	0.220
M18 HBV DNA <2000 IU/mL	13/29	44.8%	19/34	55.9%	0.382
M18 HBV DNA negative	0/29	0%	3/34	8.8%	0.167

M6: 6 months after the start of the treatment, M12: end of treatment, and M18: 6 months after the end of the treatment.

**Table 4:** HBV DNA during treatment and followup, real-world cohort.

	Roma		Non-Roma		*p*
M6 HBV DNA <2000 IU/mL	30/43	69.8%	38/46	82.6%	0.154
M6 HBV DNA negative	3/43	7.0%	7/46	15.2%	0.318
M12 HBV DNA <2000 IU/mL	22/43	51.2%	37/46	80.4%	0.003
M12 HBV DNA negative	3/43	7.0%	9/46	19.6%	0.082
M18 HBV DNA <2000 IU/mL	13/43	30.2%	19/46	41.3%	0.277
M18 HBV DNA negative	0/43	0%	3/46	6.5%	0.242

M6: 6 months after the start of the treatment, M12: end of treatment, and M18: 6 months after the end of the treatment.

**Table 5:** Compliance.

	Roma		Non-Roma		*p*
Uncompleted treatment (48 weeks)	9/43	20.9%	0/46	0%	0.0009
Loss to followup at M18	14/43	32.6%	12/46	26.1%	0.502

## Abbreviations

CHB: Chronic hepatitis B
EoT: End of treatment
PEG IFN: Pegylated interferon alpha 2a.
